# Safety Assessment of the Ethanolic Seed Extract of *Mucuna pruriens* var. *pruriens*: Acute and Chronic Oral Toxicity Studies in Sprague–Dawley Rats

**DOI:** 10.3390/ph19030421

**Published:** 2026-03-04

**Authors:** Supaporn Intatham, Kanjana Jaijoy, Sunee Chansakaow, Seewaboon Sireeratawong

**Affiliations:** 1 Clinical Research Center for Food and Herbal Product Trials and Development (CR-FAH), Faculty of Medicine, Chiang Mai University, Chiang Mai 50200, Thailand; intatham_s@outlook.com; 2 Department of Pharmacology, Faculty of Medicine, Chiang Mai University, Chiang Mai 50200, Thailand; 3 McCormick Faculty of Nursing, Payap University, Chiang Mai 50000, Thailand; joi.kanjana@gmail.com; 4 Department of Pharmaceutical Sciences, Faculty of Pharmacy, Chiang Mai University, Chiang Mai 50200, Thailand; sunee.c@cmu.ac.th

**Keywords:** *Mucuna pruriens* (Linn.) DC. var. *pruriens*, acute oral toxicity, chronic oral toxicity, repeated-dose toxicity, safety evaluation, Sprague–Dawley rats

## Abstract

**Background/Objectives**: *Mucuna pruriens* (Linn.) DC. var. *pruriens* is a leguminous plant whose seeds have been used in traditional medicine, including for the enhancement of sexual function. However, scientific evidence regarding its toxicological safety remains limited. Accordingly, the present study aimed to investigate the acute and chronic oral toxicity of the ethanolic seed extract of *M. pruriens* var. *pruriens* in Sprague–Dawley rats. **Methods**: Acute oral toxicity was assessed in female rats following a single oral administration of the ethanolic seed extract of *M. pruriens* var. *pruriens* at a dose of 5000 mg/kg body weight, with animals monitored for general behavior, clinical signs, and mortality over a 14-day period. Chronic oral toxicity was evaluated in female and male rats administered the ethanolic seed extract of *M. pruriens* var. *pruriens* at doses of 100, 500, and 2500 mg/kg body weight daily for 270 days. Animals were monitored for general behavior, clinical signs, and health status throughout the study. Hematological, blood chemistry, gross pathological, and histopathological assessments were conducted at study termination. **Results**: In the acute oral toxicity study, no mortality or treatment-related behavioral abnormalities or clinical signs were observed in female rats receiving the ethanolic seed extract of *M. pruriens* var. *pruriens*, and findings were comparable to those of the control group. In the chronic oral toxicity study, no mortality occurred in any treatment group. Although statistically significant increases or decreases were observed in certain body weight, organ weight, hematological, and blood biochemical parameters compared with the control group, all values remained within established reference ranges. When considered together with the absence of abnormal behavioral changes, clinical signs, and gross pathological or histopathological alterations in major organs, these findings indicate that long-term oral administration of the ethanolic seed extract of *M. pruriens* var. *pruriens* did not result in chronic toxicity. **Conclusions**: The ethanolic seed extract of *M. pruriens* var. *pruriens* did not produce acute or chronic oral toxicity in Sprague–Dawley rats. Nevertheless, further clinical investigations are recommended to confirm its long-term safety for human use.

## 1. Introduction

A leguminous plant belonging to the family Fabaceae (Leguminosae), *M. pruriens* (Linn.) DC. var. *pruriens* is native to Africa and Asia, where it is naturally distributed across tropical and subtropical regions of both continents. Owing to its extensive agricultural and medicinal uses, this species has been introduced and cultivated in various other parts of the world, including North America, Central America, South America, the Pacific Islands, and certain regions of Australia. As a result, *M. pruriens* var. *pruriens* is now widely distributed throughout tropical regions worldwide [1]. This plant, commonly known in Thai as “Mha-Mui,” is widely recognized for its characteristic hairy pods [2]. These pods are densely covered with stinging trichomes, which are fine, needle-like hairs containing irritant compounds such as mucunain [3]. Contact with the trichomes can cause skin irritation characterized by intense itching, erythematous wheals resembling urticaria, pain, swelling, and, in some cases, inflammation and a burning sensation [4]. Consequently, the plant is often regarded by the public as an undesirable species to be eliminated. Nevertheless, *M. pruriens* var. *pruriens* has long been used as a traditional medicinal herb, particularly in India, where its seeds are processed and marketed as dietary supplements [5]. The seeds possess sedative properties but can be toxic when consumed in excessive amounts. Moreover, ingestion of raw or unprocessed seeds without roasting, steaming, or cooking may lead to adverse effects such as nausea, vomiting, abdominal pain, or hallucinations [6].

Previous studies have reported the pharmacological activities of *M. pruriens* var. *pruriens* seeds containing L-DOPA, including the enhancement of male sexual performance [7], antioxidant properties [8], anti-inflammatory effect [9], anti-anxiety and anti-depressive effects [10], and anti-Parkinson activity [11]. Based on these pharmacological properties, Sao Hai Hospital in Saraburi Province, Thailand, has used *M. pruriens* var. *pruriens* seeds to relieve pain associated with arthritis and erectile dysfunction. However, scientific evidence confirming the safety of this herbal medicine remains limited. Consequently, evaluating the safety of *M. pruriens* var. *pruriens* extracts found in Thailand is considered essential to provide scientific evidence supporting the safe use and further development of this plant for preventive and/or therapeutic purposes.

Toxicological evaluation is an essential process for establishing the safety profile of herbal medicines intended for therapeutic use [12]. Acute toxicity testing is performed to determine the adverse effects resulting from a single oral administration of a test substance in experimental animals [13]. According to the Organisation for Economic Co-operation and Development (OECD) Test Guideline and the World Health Organization (WHO) guideline, the fixed-dose procedure is employed to estimate the median lethal dose (LD_50_) and identify the dose producing evident but non-lethal toxic effects [14,15]. Experimental animals are carefully monitored for behavioral changes, clinical signs, and mortality for 14 days following administration. Although no immediate toxicity may be observed, certain substances may exert delayed or cumulative effects, highlighting the need for long-term evaluation [16]. In accordance with the OECD Test Guideline and the WHO guideline, chronic toxicity testing is conducted to assess the potential toxic effects of repeated exposure over an extended duration, typically ranging from six to twelve months [15,17]. This assessment is used to identify systemic and target-organ toxicity through neurological, biochemical, hematological, and histopathological examinations, and to determine whether the observed toxic responses are reversible after treatment cessation. Such comprehensive toxicological investigations provide fundamental data to ensure the long-term safety of herbal medicines [18].

Only one previous study has reported the subacute toxicity of an aqueous extract prepared from the seeds of *M. pruriens* var. *pruriens*. In that study, the extract was administered orally to rats by gavage at a dose of 300 mg/kg for 14 consecutive days. The results showed no evidence of toxicity in internal organs, and no histopathological lesions were observed at this dose within the short-term exposure period [19]. However, toxicological data on seed extracts of *M. pruriens* var. *pruriens* remain limited, and no studies have yet investigated the chronic toxicity of this plant. Therefore, it is of great importance to conduct a comprehensive toxicological evaluation of the standardized ethanolic seed extract of *M. pruriens* var. *pruriens*, the variety commonly found in Thailand. The present study was designed to assess the acute and chronic oral toxicity of the standardized extract of *M. pruriens* var. *pruriens* in experimental animals, generating scientific evidence to support its safety prior to potential future clinical studies in humans.

## 2. Results

### 2.1. Quality Assessment of the Raw Material

The roasted seeds of *M. pruriens* var. *pruriens* complied with the preliminary internal quality standards established for this study, as presented in Table 1.

**Figure 1:**
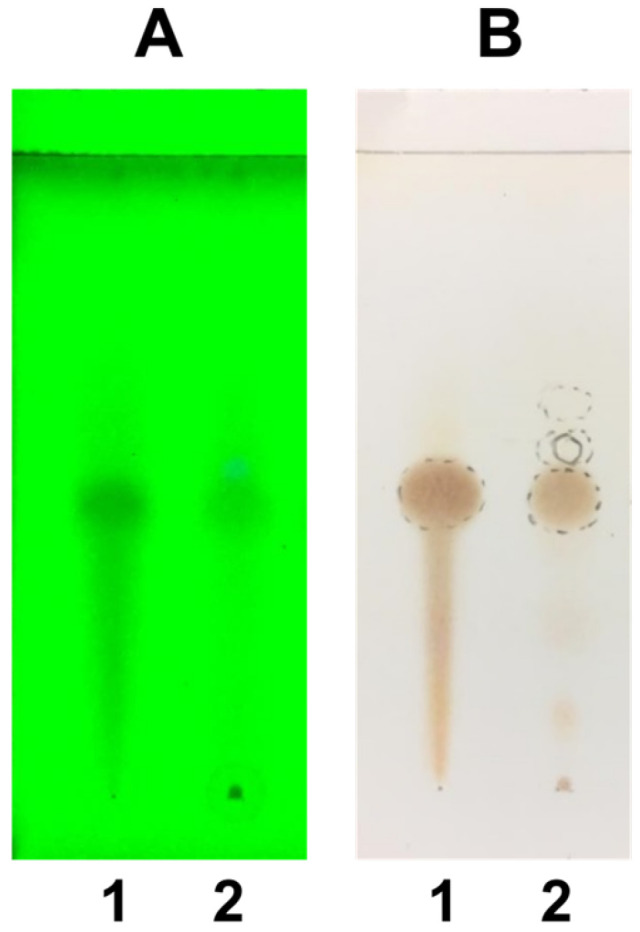
TLC chromatogram of *M. pruriens* var. *pruriens.* The stationary phase was silica gel GF254; The mobile phase consisted of *n*-butanol:acetic acid:water (4:1:1); detected with UV 254 nm (**A**); spraying with ninhydrin spraying reagent (**B**); Spot 1 = standard L-DOPA; Spot 2 = seed extract of *M. pruriens* var. *pruriens*.

Thin-layer chromatography (TLC) analysis confirmed the presence of L-DOPA in the crude extract of the raw material. The standard L-DOPA exhibited an Rf value of 0.46 (brown spot with ninhydrin). The *M. pruriens* var. *pruriens* seed extract showed a corresponding spot at Rf 0.44, alongside other phytochemical constituents at Rf values 0.12, 0.16, 0.52, and 0.60 (Figure 1).

### 2.2. Quality Assessment of the Plant Extract

The powdered seeds of *M. pruriens* var. *pruriens* were extracted with continuous extraction using 80% ethanol as the solvent, then concentrated with a rotary evaporator, yielding a dark brown syrupy mass with a yield of 12.28% *w/w*. The quality control of the extract was conducted using high-performance liquid chromatography (HPLC) and compact mass spectrometry (CMS).

**Figure 2:**
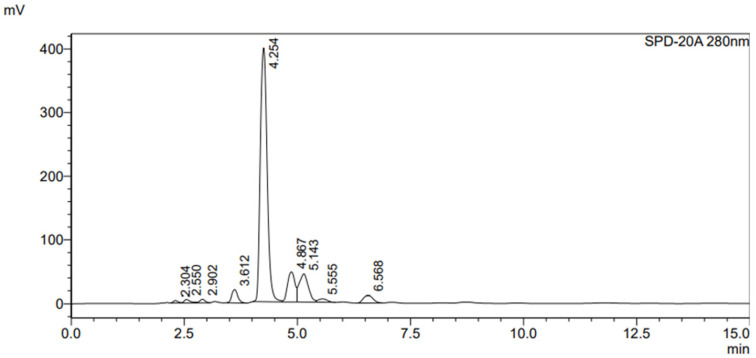
HPLC chromatogram of the ethanolic seed extract of *M. pruriens* var. *pruriens*.

The HPLC chromatogram of the ethanolic seed extract of *M. pruriens* var. *pruriens* (Figure 2) showed a prominent peak at a retention time of approximately 4.3 min, identifying L-DOPA as the major constituent (0.54 mg/mL). Minor peaks appeared at 3.6, 4.9, 5.1, and 6.6 min.

**Figure 3:**
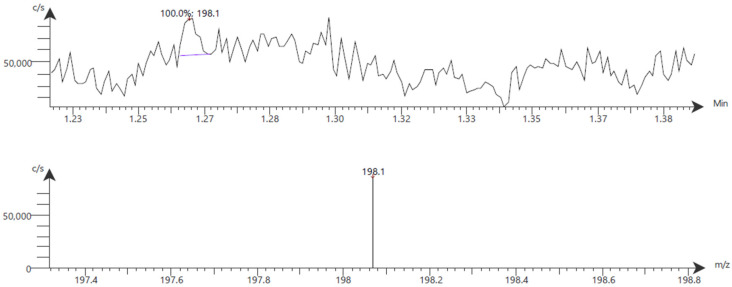
APCI mass spectrum of the ethanolic seed extract of *M. pruriens* var. *pruriens*, with positive-ion scan mode at 10–1200 *m*/*z*.

**Figure 4:**
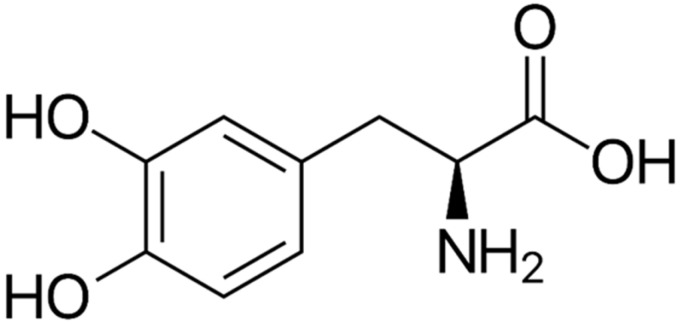
Chemical structure of L-DOPA.

Compounds in the ethanolic seed extract of *M. pruriens* var. *pruriens* were analyzed using CMS in selected ion monitoring (SIM) mode with atmospheric pressure chemical ionization (APCI) in positive-ion mode. L-DOPA, used as a biomarker, was detected in the sample, confirming its presence and contribution to the therapeutic profile of *M. pruriens* var. *pruriens*, as shown in Figure 3 and Figure 4.

### 2.3. Findings of the Acute Oral Toxicity Evaluation

In the sighting study for acute oral toxicity, a single female rat was orally administered the ethanolic seed extract of *M. pruriens* var. *pruriens* at a dose of 2000 mg/kg body weight. During the first 24 h after administration, no abnormal general behavioral changes or clinical signs were observed, as indicated by the Hippocratic screening test. Additionally, no mortality was recorded during the observation period. These findings are summarized in Table S1.

The acute oral toxicity evaluation showed that a single oral administration of the ethanolic seed extract of *M. pruriens* var. *pruriens* at a dose of 5000 mg/kg body weight to female rats did not produce any abnormal general behavioral changes or clinical signs within the first 24 h after dosing. The feeding behavior, water consumption, and excretory patterns of the treated rats were comparable to those of the control group. Furthermore, no mortality was observed in any of the experimental groups during the 14-day observation period.

**Figure 5:**
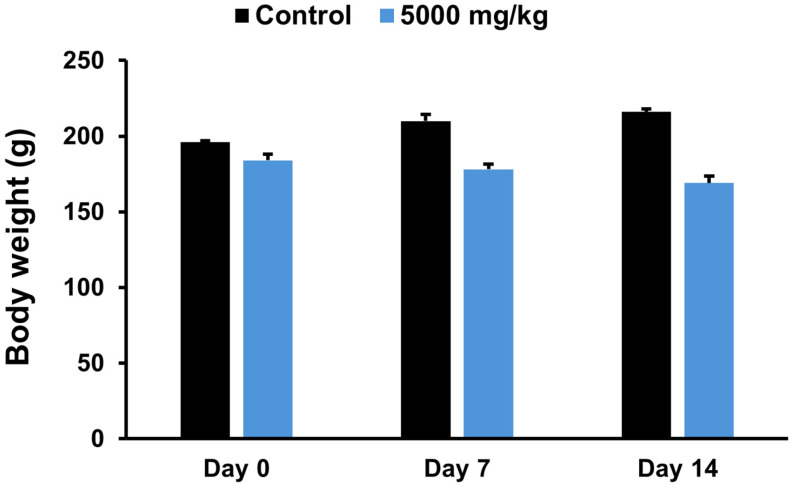
Effects of acute oral administration of the ethanolic seed extract of *M. pruriens* var. *pruriens* on body weight (g) in female rats. All values are reported as mean ± S.E.M. (*n* = 5 per group).

Body weight changes in female rats before treatment and on days 7 and 14 are presented in Figure 5. The body weight of rats in the control group showed a gradual increase over time, whereas rats receiving the ethanolic seed extract of *M. pruriens* var. *pruriens* exhibited a decreasing trend in body weight during the observation period. However, no statistically significant differences in body weight were observed between the treated group and the control group at any time point.

**Figure 6:**
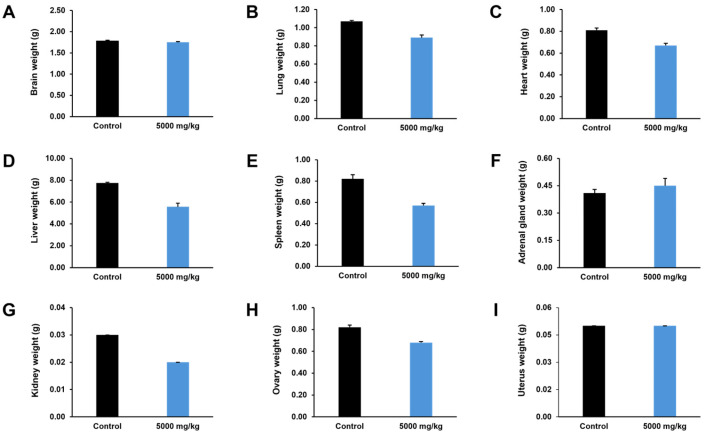
Effects of acute oral administration of the ethanolic seed extract of *M. pruriens* var. *pruriens* on organ weight (g) in female rats. (**A**) Brain weight. (**B**) Lung weight. (**C**) Heart weight. (**D**) Liver weight. (**E**) Spleen weight. (**F**) Adrenal gland weight. (**G**) Kidney weight. (**H**) Ovary weight. (**I**) Uterus weight. All values are reported as mean ± S.E.M. (*n* = 5 per group).

The effects of the ethanolic seed extract of *M. pruriens* var. *pruriens* on organ weights, including the brain, lungs, heart, liver, spleen, adrenal glands, ovaries, and uterus of female rats, are shown in Figure 6. No statistically significant differences in organ weights were detected between rats treated with the ethanolic seed extract of *M. pruriens* var. *pruriens* and the control group. In addition, gross examination of the internal organs revealed no macroscopic abnormalities in the treated group compared with the control group.

### 2.4. Findings of the Chronic Oral Toxicity Evaluation

During long-term daily oral administration of the ethanolic seed extract of *M. pruriens* var. *pruriens* for 270 days, both male and female rats were routinely examined for treatment-related effects. The Hippocratic screening test demonstrated the absence of abnormal behavioral changes or clinical signs in rats receiving the ethanolic seed extract of *M. pruriens* var. *pruriens* at all tested dose levels compared with the control group (Table S2). Food and water intake, as well as excretory patterns, were similar to those of the control group. Moreover, no treatment-related changes were noted in skin and fur condition, ocular appearance, respiratory pattern, mucous membranes, peripheral circulation of the ears and extremities, or autonomic and central nervous system function. Importantly, no mortality occurred in any group throughout the entire experimental period.

**Figure 7:**
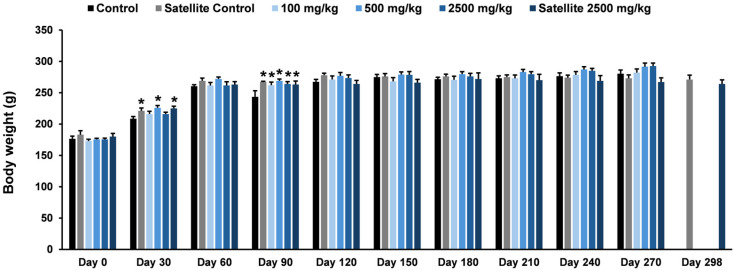
Effects of chronic oral administration of the ethanolic seed extract of *M. pruriens* var. *pruriens* on body weight (g) in female rats. All values are reported as mean ± S.E.M. (*n* = 10 per group; *n* = 5 per group for satellite groups). * *p* < 0.05 in comparison to the control group.

**Figure 8:**
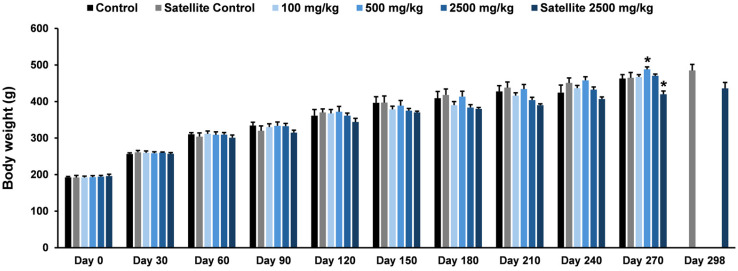
Effects of chronic oral administration of the ethanolic seed extract of *M. pruriens* var. *pruriens* on body weight (g) in male rats. All values are reported as mean ± S.E.M. (*n* = 10 per group; *n* = 5 per group for satellite groups). * *p* < 0.05 in comparison to the control group.

The effects of the ethanolic seed extract of *M. pruriens* var. *pruriens* on body weight in female and male rats are presented in Figure 7 and Figure 8, respectively. In female rats, a statistically significant increase in body weight was observed on Day 30 in the satellite control group, the treatment group receiving the ethanolic seed extract of *M. pruriens* var. *pruriens* at a dose of 500 mg/kg body weight, and the satellite treatment group compared with the control group. On Day 90 of treatment, female rats in the satellite control group, all dose groups receiving the ethanolic seed extract of *M. pruriens* var. *pruriens*, and the satellite treatment group exhibited a statistically significant increase in body weight compared with the control group. In male rats, body weight was statistically significantly increased in the 500 mg/kg treatment group on the final day of treatment, whereas a statistically significant decrease was observed in the satellite treatment group compared with the control group.

**Figure 9:**
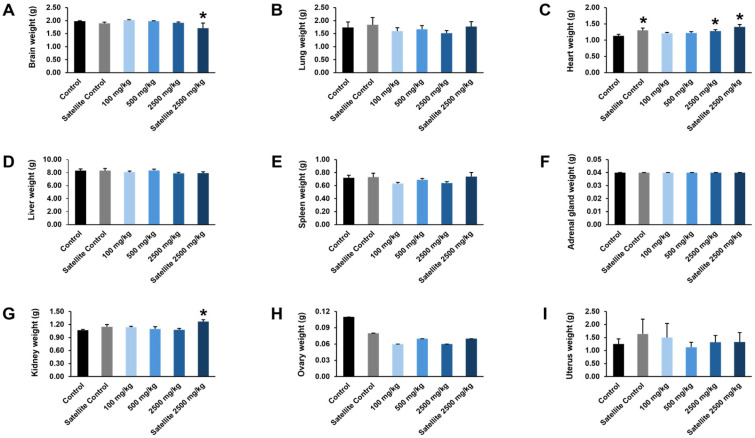
Effects of chronic oral administration of the ethanolic seed extract of *M. pruriens* var. *pruriens* on organ weight (g) in female rats. (**A**) Brain weight. (**B**) Lung weight. (**C**) Heart weight. (**D**) Liver weight. (**E**) Spleen weight. (**F**) Adrenal gland weight. (**G**) Kidney weight. (**H**) Ovary weight. (**I**) Uterus weight. All values are reported as mean ± S.E.M. (*n* = 10 per group; *n* = 5 per group for satellite groups). * *p* < 0.05 in comparison to the control group.

**Figure 10:**
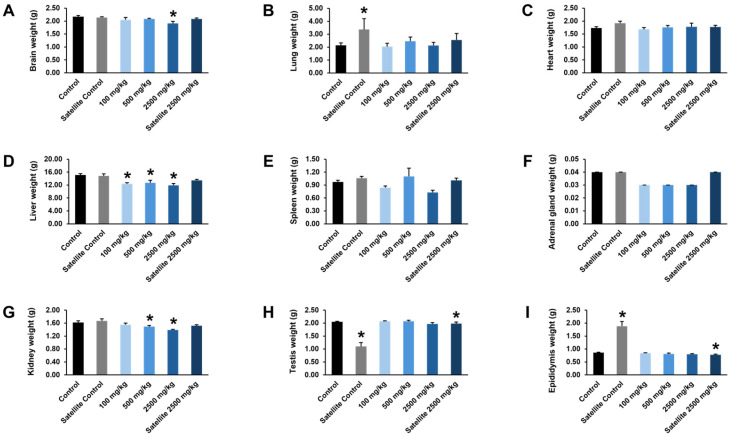
Effects of chronic oral administration of the ethanolic seed extract of *M. pruriens* var. *pruriens* on organ weight (g) in male rats. (**A**) Brain weight. (**B**) Lung weight. (**C**) Heart weight. (**D**) Liver weight. (**E**) Spleen weight. (**F**) Adrenal gland weight. (**G**) Kidney weight. (**H**) Testis weight. (**I**) Epididymis weight. All values are reported as mean ± S.E.M. (*n* = 10 per group; *n* = 5 per group for satellite groups). * *p* < 0.05 in comparison to the control group.

At the end of the chronic oral toxicity study, the effects of the ethanolic seed extract of *M. pruriens* var. *pruriens* on organ weights were evaluated in both female and male rats. In female rats, a statistically significant increase in heart weight was observed in the satellite control group and the group receiving the ethanolic seed extract of *M. pruriens* var. *pruriens* at a dose of 2500 mg/kg body weight compared with the control group. In the satellite treatment group, a statistically significant decrease in brain weight was noted, whereas heart and kidney weights were significantly increased relative to the control group (Figure 9). In male rats, comparison with the control group revealed a statistically significant increase in lung and epididymis weights and a significant decrease in testes weight in the satellite control group. In the group receiving the ethanolic seed extract of *M. pruriens* var. *pruriens* at a dose of 100 mg/kg body weight, liver weight was significantly decreased. Male rats treated with 500 mg/kg body weight of the ethanolic seed extract of *M. pruriens* var. *pruriens* showed statistically significant reductions in liver and kidney weights. In the group receiving 2500 mg/kg body weight of the ethanolic seed extract of *M. pruriens* var. *pruriens*, statistically significant decreases in brain, liver, and kidney weights were observed. Furthermore, the satellite treatment group exhibited statistically significant decreases in testes and epididymis weights compared with the control group (Figure 10). Therefore, the toxicological significance of these organ weight changes should be further evaluated in conjunction with gross pathological and histopathological findings.

The hematological parameters of female and male rats are summarized in Table 2 and Table 3, respectively. In female rats, no statistically significant differences in hematological values were observed in any treatment group compared with the control group. In contrast, in male rats, a statistically significant reduction in mean corpuscular hemoglobin concentration (MCHC) was detected in the groups receiving the ethanolic seed extract of *M. pruriens* var. *pruriens* at doses of 500 and 2500 mg/kg body weight, as well as in the satellite treatment group, compared with the control group.

The WBC differential counts of female rats are presented in Table 4. A statistically significant increase in white blood cell (WBC) and neutrophil (NEU) counts was observed in the satellite treatment group compared with the control group. Table 5 summarizes the WBC differential counts of male rats, showing a statistically significant decrease in lymphocyte (LYMP) counts in the groups receiving the ethanolic seed extract of *M. pruriens* var. *pruriens* at doses of 100 and 500 mg/kg body weight compared with the control group.

Blood chemistry parameters in female rats in the chronic oral toxicity study revealed that the satellite control group exhibited statistically significant increases in blood urea nitrogen (BUN) and total protein levels, accompanied by significant decreases in creatinine and total bilirubin levels compared with the control group. In the group receiving the ethanolic seed extract of *M. pruriens* var. *pruriens* at a dose of 100 mg/kg body weight, a statistically significant elevation in aspartate aminotransferase (AST) was observed. Additionally, the satellite treatment group showed a statistically significant increase in BUN levels, together with significant reductions in creatinine and total bilirubin levels relative to the control group (Table 6). In male rats, blood chemistry analysis demonstrated a statistically significant decrease in BUN levels across all treated groups compared with the control group (Table 7).

**Figure 11:**
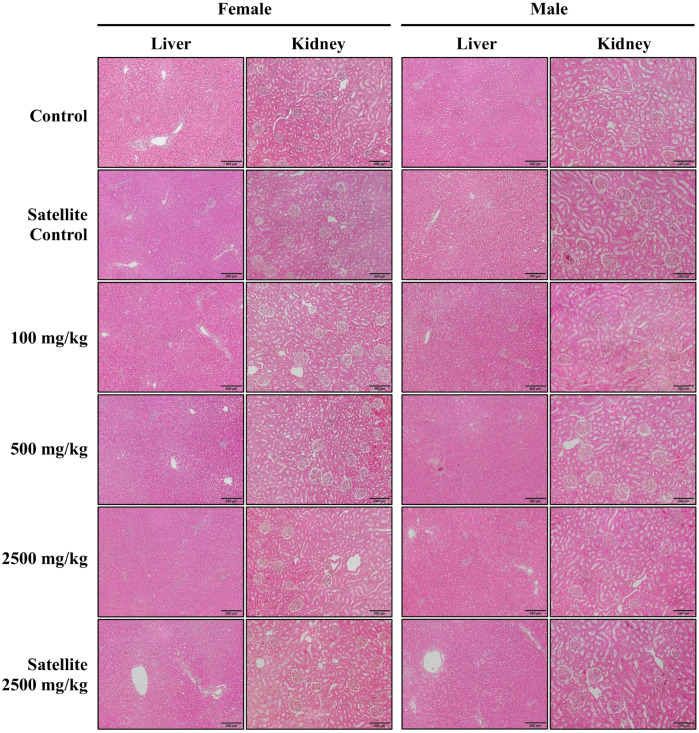
Representative histological sections of the liver and kidney from female and male rats in the chronic oral toxicity study of the ethanolic seed extract of *M. pruriens* var. *pruriens* using H&E staining. Scale bars represent 200 µm at ×10 magnification.

Gross pathological examination and macroscopic observation of internal organs from both female and male rats administered the ethanolic seed extract of *M. pruriens* var. *pruriens* at all tested dose levels revealed no treatment-related abnormalities. The examined organs, including the brain, lungs, heart, liver, spleen, adrenal glands, ovaries, uterus, testes, epididymis, pancreas, stomach, intestines, eyes, muscles, and nerves, showed no alterations in size, morphology, or color compared with the control group. Histopathological evaluation of internal organs further demonstrated the absence of treatment-related tissue damage. Brain tissue showed no neuronal cell death, and lung tissues exhibited normal pulmonary architecture without evidence of hemorrhage or obstructive exudates. Cardiovascular tissues revealed no signs of inflammation or necrosis, while hepatic tissues displayed normal architecture without hepatocellular degeneration. No pathological alterations were observed in splenic or pancreatic tissues. Endocrine tissues, including the adrenal cortex and medulla, were clearly distinguishable. Renal tissues displayed normal morphology, and reproductive tissues showed no histopathological abnormalities. Histopathological images of vital organs, specifically the liver and kidney, from both female and male rats are shown in Figure 11.

## 3. Discussion

*M. pruriens* var. *pruriens* is commonly recognized for its undesirable effect of causing skin irritation or itching, which is primarily attributed to its alkaloid constituents, and despite this unfavorable characteristic, the plant has been traditionally utilized as a medicinal herb in specific health-related contexts in Thailand [7,20]. Beyond its traditional use, this plant has become the subject of growing scientific investigation, with reported pharmacological activities involving the nervous and endocrine systems [8,21,22]. However, although existing studies have predominantly focused on its therapeutic potential, relatively few investigations have evaluated the toxicological effects of single and repeated exposure under controlled experimental conditions, highlighting the need for systematic safety assessments conducted in accordance with established standard guidelines. In addition, variability in phytochemical composition arising from differences in plant origin, extraction procedures, and standardization strategies has been recognized as an important factor influencing both efficacy and safety profiles [23,24]. Accordingly, the present study was designed to evaluate the acute and chronic oral toxicity of a standardized extract of *M. pruriens* var. *pruriens* in experimental animals using standardized protocols consistent with internationally accepted guidelines.

This study establishes a quality profile for roasted *M. pruriens* var. *pruriens* seeds and their 80% ethanolic extracts, addressing the lack of official standards in the THP. Using THP-based protocols, we defined preliminary internal standards to ensure pharmacological consistency in raw materials for future product development. Chromatographic profiling by TLC and HPLC confirmed both qualitatively and quantitatively that L-DOPA is the primary bioactive marker. TLC analysis revealed a correlation between the seed extract of *M. pruriens* var. *pruriens* and the standard (Rf 0.44). The appearance of characteristic brown spots after ninhydrin visualization indicates a rich amino acid profile. HPLC enabled a more detailed characterization of the ethanolic seed extract of *M. pruriens* var. *pruriens*’s composition. The major peak at approximately 4.3 min was unequivocally identified as L-DOPA. Minor peaks at retention times of 3.6, 4.9, 5.1, and 6.6 min suggest the presence of additional secondary metabolites within a complex phytochemical matrix. To corroborate these results, CMS in APCI positive-ion mode was utilized, which confirmed the detection of the biomarker and supported the therapeutic potential of the ethanolic seed extract of *M. pruriens* var. *pruriens*.

The acute and chronic oral toxicity studies of the ethanolic seed extract of *M. pruriens* var. *pruriens* in rats were carried out to evaluate its safety and to identify suitable dose levels for potential use in future clinical studies [25]. The assessment relied on careful and systematic health monitoring of the animals before treatment and throughout the study period. Observations included evaluations of respiratory function, gastrointestinal and excretory activity, neurological responses, musculoskeletal condition, and general behavior [14,15,17]. Additionally, regular health surveillance and appropriate control of housing and environmental conditions were maintained to ensure the reliability of the toxicity evaluation [26].

Acute oral toxicity study is a critical first step in toxicological evaluation, providing early information on potential adverse effects after short-term or single-dose exposure [13]. It supports the identification of animal tolerance and selection of appropriate dose levels, forming a basis for subsequent long-term toxicity assessments [14,27]. The acute oral toxicity assessment of the ethanolic seed extract of *M. pruriens* var. *pruriens* was initiated using a stepwise approach. A single female rat was first administered the ethanolic seed extract of *M. pruriens* var. *pruriens* at a dose of 2000 mg/kg body weight, followed by a preliminary qualitative evaluation of the general toxicological profile using a Hippocratic screening test [14,15,28]. During the 24 h observation period, no abnormalities were detected in behavioral, neurological, autonomic, or physical responses, and no mortality was observed. Based on these findings, a higher dose of 5000 mg/kg body weight was subsequently administered to female rats and compared with a control group receiving distilled water. Throughout the 14-day observation period, female rats treated with the ethanolic seed extract of *M. pruriens* var. *pruriens* showed no treatment-related general behavioral or clinical signs, and body weight changes were comparable to those of the control group. Moreover, no mortality occurred. Gross pathological examination performed on Day 15 revealed no abnormalities in internal organs, and organ weights did not differ from those of the control group. Taken together, the absence of adverse behavioral and clinical signs, mortality, or gross pathological changes demonstrates that a single oral administration of the ethanolic seed extract of *M. pruriens* var. *pruriens* at a dose of 5000 mg/kg body weight did not induce acute oral toxicity in female rats. However, the absence of acute toxicity does not preclude potential adverse effects associated with repeated or long-term exposure. Therefore, chronic oral toxicity evaluation is required to assess cumulative or delayed toxic effects and to support long-term safety assessment and human risk evaluation.

Chronic oral toxicity study is essential for evaluating the effects of repeated exposure to a test substance over prolonged periods, enabling the assessment of both persistent and potentially reversible toxic effects [17]. Such a study provides critical data for human health risk assessment by identifying dose levels that do not cause adverse effects, thereby supporting the establishment of safe exposure limits for human use [29]. In the present investigation, a chronic oral toxicity study of the ethanolic seed extract of *M. pruriens* var. *pruriens* was evaluated in male and female rats administered daily doses of 100, 500, and 2500 mg/kg body weight for 270 days. Animal health was monitored continuously throughout the exposure period, with functional status assessed using the Hippocratic screening test. This assessment included evaluation of motor activity to assess neuromuscular function, respiratory rate to evaluate respiratory function, the righting reflex to examine neurological integrity and coordination, and screen grip to assess muscle strength and neuromuscular performance [15,17,28]. No adverse clinical signs related to test substance exposure or behavioral alterations were observed in any dose group during the treatment phase or the subsequent 28-day recovery period, indicating that the ethanolic seed extract of *M. pruriens* var. *pruriens* did not adversely affect these physiological and neurological systems. Furthermore, no mortality occurred throughout the study.

Body weight monitoring and organ weight measurement are key parameters in a chronic oral toxicity study. Body weight reflects the general health status and long-term physiological response of experimental animals to the test substance, whereas changes in organ weight may serve as early indicators of physiological alterations or target-organ toxicity before overt pathological lesions become evident [30,31]. Together, these parameters provide essential support for interpreting experimental outcomes and assessing the long-term safety of the test substance. In the present chronic oral toxicity study, body weights of both male and female rats were monitored throughout the experimental period, and final body and organ weights were measured at study termination for comparison with the control group. Changes in body weight and certain organ weights were observed in rats of both sexes receiving the ethanolic seed extract of *M. pruriens* var. *pruriens*. Nonetheless, in the absence of treatment-related behavioral abnormalities or clinical signs of toxicity, together with the lack of gross pathological and histopathological alterations, these changes are more likely attributable to normal biological variability rather than direct effects of the test substance, as body and organ weights can be influenced by multiple factors, including age, sex, growth stage, physiological condition, and inter-individual differences [17,32,33]. Consequently, the ethanolic seed extract of *M. pruriens* var. *pruriens* was not considered to induce toxicologically relevant alterations in body weight or organ weight.

The evaluation of hematological parameters, along with WBC differential counts, represents an important component of the chronic oral toxicity study, as these measures reflect the physiological status and homeostasis of the hematopoietic and immune systems in experimental animals. Alterations in red blood cell (RBC)- related parameters may indicate disturbances in hematopoiesis, whereas changes in the number and distribution of WBC populations can reflect systemic responses and modulation of immune function [34,35]. Furthermore, platelet parameters are closely linked to blood coagulation mechanisms [36]. The results of the present study indicated that hematological profiles in female rats receiving the ethanolic seed extract of *M. pruriens* var. *pruriens* were not markedly affected by treatment and remained comparable to those of the control group. In contrast, male rats treated with the ethanolic seed extract of *M. pruriens* var. *pruriens* exhibited a statistically significant reduction in MCHC compared with the control group. In addition, analysis of WBC differential counts in both sexes revealed statistically significant differences in certain parameters, including WBC count, NEU count, and LYMP count, following administration of the ethanolic seed extract of *M. pruriens* var. *pruriens*. Nevertheless, when these statistically significant alterations in hematological parameters and WBC differential counts were evaluated in relation to established reference ranges, all values were found to remain within the normal reference range [37]. Accordingly, these findings suggest that the ethanolic seed extract of *M. pruriens* var. *pruriens* does not exert toxicologically relevant effects on the hematological system.

Blood chemistry analysis plays a crucial role in evaluating chronic oral toxicity, as it provides valuable insights into the function of internal organs and physiological homeostasis in experimental animals. In particular, the liver and kidneys are primary organs involved in metabolism and the elimination of xenobiotics [38,39]. Renal function-related parameters, such as BUN and creatinine, are commonly used as indicators of excretory capacity, whereas liver function parameters, including AST, alanine aminotransferase (ALT), alkaline phosphatase (ALP), total protein, albumin, and bilirubin, reflect hepatocellular integrity as well as protein and metabolic balance [40,41]. Evaluation of these blood chemistry parameters, therefore, supports a comprehensive interpretation of experimental findings and contributes to the assessment of the long-term safety of the test substance. The results of the present study showed that administration of the ethanolic seed extract of *M. pruriens* var. *pruriens* led to statistically significant increases or decreases in certain blood chemistry parameters in both female and male rats compared with the control group, including BUN, creatinine, total protein, total bilirubin, and AST. However, when interpreted in the context of established reference intervals, all altered parameters remained within normal reference ranges [42,43,44,45]. Therefore, these changes do not indicate impairment of renal or hepatic function, suggesting that the ethanolic seed extract of *M. pruriens* var. *pruriens* did not induce toxicologically relevant effects on kidney or liver function.

Gross pathological examination and macroscopic observation, combined with histopathological evaluation, are essential components of the chronic oral toxicity study, as they enable the assessment of structural alterations in internal organs that may occur from prolonged exposure to a test substance. Macroscopic examination facilitates the detection of gross abnormalities at the organ level, including changes in organ size, shape, and color [46]. Whereas histopathological evaluation provides confirmation of tissue-level alterations that may not be evident from gross inspection alone [47]. In the present study, gross pathological examination of internal organs from both female and male rats administered the ethanolic seed extract of *M. pruriens* var. *pruriens* revealed no abnormalities in organ size, morphology, or coloration when compared with the control group. Furthermore, histopathological assessment showed no structural tissue alterations or evidence of tissue damage attributable to the test substance. These findings support the conclusion that, under the dose levels and exposure duration employed in this study, the ethanolic seed extract of *M. pruriens* var. *pruriens* did not induce structural toxicity in the internal organs of experimental animals.

## 4. Materials and Methods

### 4.1. Raw Material Preparation and Selection

**Figure 12:**
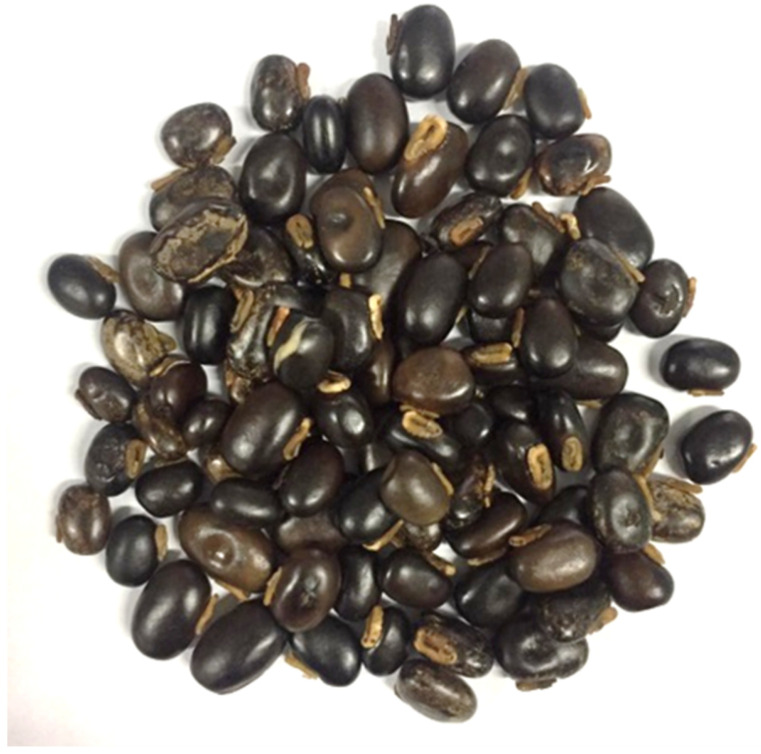
Seeds of *M. pruriens* var. *pruriens*.

Roasted seeds of *M. pruriens* var. *pruriens* were obtained from the Im-Boon-Jung Foundation at Sao Hai Hospital in Saraburi, Thailand (Figure 12). Seeds were selected based on integrity and uniform color, while over-roasted or charred seeds were excluded. Prior to extraction, the seeds were coarsely ground to reduce their size.

### 4.2. Quality Evaluation of Raw Materials

Quality assessment was conducted in accordance with the guidelines established by the Thai Herbal Pharmacopoeia (THP) [48]. The evaluated parameters were as follows:

- Physical and chemical properties included loss on drying, total ash, acid-insoluble ash, and extractive values using 95% ethanol and chloroform-saturated water.
- TLC was performed on Silica gel GF254 plates using *n*-butanol:acetic acid:water (4:1:1) as the mobile phase. Detection was carried out under UV light at 254 nm and 366 nm, as well as with ninhydrin spraying reagent.

### 4.3. Preparation of the Plant Extract

The seed powder of *M. pruriens* var. *pruriens* was extracted using 80% ethanol at a solid-to-solvent ratio of 1:6 (*w*/*v*) in a Soxhlet extractor for 8 h over 3 cycles. The extract was then concentrated using a rotary evaporator to yield a dark brown, syrupy mass. For safety evaluation in experimental animals, the extract was dissolved in distilled water to achieve the required dose concentrations and administered orally to rats at a dosing volume not exceeding 2 mL/kg body weight.

### 4.4. Phytochemical Analysis by HPLC

#### 4.4.1. Preparation of Standard and Test Solutions

For quantitative analysis, a L-DOPA standard stock solution was prepared at 1 mg/mL by dissolving 25 mg of standard in 25 mL of 0.1 N formic acid. This solution was diluted to prepare a calibration curve spanning 0.02–0.8 mg/mL and then filtered through a 0.45 μm nylon syringe filter. The test sample was prepared at a concentration of 5 mg/mL in 0.1 N formic acid and filtered through a 0.22 μm nylon syringe filter before injection.

#### 4.4.2. Chromatographic Conditions

Chemical profiling was performed on an HPLC Shimadzu LC20AD system (SHIMADZU, Kyoto, Japan) with an RP-18 GP column (250 × 4.6 mm, 5 µm). The mobile phase was 0.1 N formic acid and methanol (98:2), at a flow rate of 1 mL/min, with UV detection at 280 nm.

### 4.5. Chemical Identification by CMS

The ethanolic seed extract of *M. pruriens* var. *pruriens* solution (0.01 mg/mL in 99% methanol, HPLC grade) was filtered using a 0.22 µm syringe filter. A 10 µL aliquot was injected into the CMS (Advion, NY, USA). Mass spectra were acquired in SIM mode with a positive APCI source at a resolution of 0.5 to 0.7 *m*/*z*.

### 4.6. Experimental Animals and Ethical Approval

Male and female Sprague–Dawley rats with body weights of 180 to 200 g were purchased from the National Laboratory Animal Center, Mahidol University, Nakhon Pathom, Thailand. The animals were maintained in an environmentally controlled animal facility at a temperature of 25 ± 1 °C and a relative humidity of 60%, under a 12 h light and 12 h dark cycle, with unrestricted access to a standard chow diet and drinking water. All animals were acclimatized for at least one week prior to the experiment. The experimental protocol was reviewed and approved by the Animal Ethical Committee of the Faculty of Medicine, Chiang Mai University, under approval number 35/2559 (7 December 2016).

### 4.7. Acute Oral Toxicity Evaluation

Following the OECD Test Guideline 420, Annex 2 (Flow chart for the sighting study) [14], and the WHO guideline [15], a single female rat was orally administered the ethanolic seed extract of *M. pruriens* var. *pruriens* at a dose of 2000 mg/kg body weight. General behavioral changes and clinical signs, including neuromuscular disturbances such as muscle spasm, gastrointestinal symptoms such as watery diarrhea and vomiting, and reduced activity manifested as sedation, were monitored after administration, with particular emphasis on the first 4 h. Additionally, a Hippocratic screening test was conducted hourly to assess motor activity, respiratory rate, righting reflex, and screen grip, with observations continued for up to 24 h after dosing, and any mortality was recorded [28].

As no mortality was observed in the sighting study, female rats were subsequently allocated to two experimental groups, each consisting of five animals, as follows:

Group 1 (Control): Distilled water (2 mL/kg body weight).

Group 2 (Treatment): The ethanolic seed extract of *M. pruriens* var. *pruriens* (5000 mg/kg body weight).

The test substance was administered orally as a single dose, and general behavioral changes and clinical signs were monitored during the first 6 h and daily for 14 days thereafter. Body weights were recorded weekly, and mortality was monitored throughout the study.

At the end of the 14-day observation period, the rats were anesthetized by intraperitoneal injection of thiopental sodium at a dose of 150 mg/kg body weight. Vital signs, reflexes, and pulse were assessed to confirm an adequate depth of anesthesia prior to further procedures [49]. Subsequently, a gross pathological examination was performed to assess the macroscopic abnormalities of the internal organs, including the brain, lungs, heart, liver, spleen, adrenal glands, ovaries, uterus, pancreas, stomach, intestines, eyes, muscles, and nerves, all of which were excised and weighed. When gross abnormalities were observed, the relevant tissues were fixed in 10% neutral buffered formalin and processed for histopathological examination using Hematoxylin and Eosin (H&E) staining.

### 4.8. Chronic Oral Toxicity Evaluation

In compliance with the OECD Test Guideline 452 [17] and the WHO Guidelines [15], female and male rats were divided into six experimental groups. Groups 1 and 3–5 consisted of 10 female and 10 male rats per group, whereas Groups 2 and 6 consisted of 5 female and 5 male rats per group, as follows:

Group 1 (Control): Distilled water (2 mL/kg body weight).

Group 2 (Satellite Control): Distilled water (2 mL/kg body weight).

Groups 3–5 (Treatment): The ethanolic seed extract of *M. pruriens* var. *pruriens* at doses of 100, 500, and 2500 mg/kg body weight, respectively.

Group 6 (Satellite Treatment): The ethanolic seed extract of *M. pruriens* var. *pruriens* at a dose of 2500 mg/kg body weight.

Rats in Groups 1 and 3–5 were treated orally once daily for 270 days, whereas rats in Groups 2 and 6 were treated for 270 days followed by a 28-day recovery period without treatment. Throughout the experimental period, general behavioral changes and clinical signs were observed, and any abnormalities were recorded. Hippocratic screening was additionally conducted to evaluate neurological and physiological parameters [28]. Body weights were recorded throughout the study period. Any mortality observed during the study was documented, and a gross pathological examination was performed to determine the cause of death.

At the end of the treatment or recovery period, the rats were anesthetized by intraperitoneal injection of thiopental sodium at a dose of 150 mg/kg body weight. Death was confirmed by assessment of vital signs, reflexes, and pulse prior to further procedures [49]. Blood samples were collected via cardiac puncture for hematological analysis, and white blood cell (WBC) differential counts using a Mindray BC-5300 Vet automated hematology analyzer (Shenzhen, China). For blood biochemical analysis, an automated BX-3010 analyzer (Sysmex, Kobe, Japan) was used. A complete gross pathological examination was conducted to assess gross pathological changes in internal organs, including the brain, lungs, heart, liver, spleen, adrenal glands, ovaries, uterus, testes, epididymis, pancreas, stomach, intestines, eyes, muscles, and nerves. All examined organs were removed and weighed, after which the collected tissues were fixed in 10% neutral buffered formalin, stained with H&E, and examined for histopathological evaluation.

### 4.9. Statistical Analysis

Experimental data were expressed as mean ± standard error of the mean (S.E.M.). For the acute oral toxicity evaluation involving two experimental groups, data distribution was assessed using the Shapiro–Wilk test. Normally distributed data were analyzed using Student’s *t*-test, whereas non-normally distributed data were analyzed using the Mann–Whitney *U* test. For the chronic oral toxicity evaluation involving more than three experimental groups, data distribution was similarly assessed using the Shapiro–Wilk test. Normally distributed data were analyzed using one-way analysis of variance (ANOVA) followed by Tukey’s post hoc test for multiple comparisons, whereas non-normally distributed data were analyzed using the Kruskal–Wallis test followed by Dunn’s test. A *p*-value of less than 0.05 was considered statistically significant. All statistical analyses were performed using SPSS Statistics software, version 25 (SPSS Inc., Chicago, IL, USA).

## 5. Conclusions

The acute and chronic oral toxicity studies of the ethanolic seed extract of *M. pruriens* var. *pruriens* in rats demonstrated that a single oral administration at a dose of up to 5000 mg/kg body weight did not induce acute toxicity. In addition, repeated oral administration at doses of 100, 500, and 2500 mg/kg body weight for 270 days did not produce treatment-related changes in external appearance, general behavior, or clinical signs in either male or female rats. Although certain hematological and blood chemistry parameters showed statistically significant alterations, these values remained within established reference ranges and were not associated with gross pathological and histopathological changes in major organs. Collectively, these findings indicate that the ethanolic seed extract of *M. pruriens* var. *pruriens* does not exert acute or long-term toxic effects in rats. Nonetheless, despite its historical use in humans, scientific evidence regarding long-term safety remains limited, and further clinical studies are warranted to confirm its safety and suitability for prolonged use in humans.

## Figures and Tables

**Table 1:** Preliminary internal quality control parameters of the roasted seeds of *M. pruriens* var. *pruriens*.

Parameter	Result (% *w/w*)	Preliminary Internal Limit
95% Ethanol-soluble extractive	0.20	Not less than 0.2%
Water-soluble extractive	1.17	Not less than 1.0%
Loss on Drying	0.80	Not exceeding 2.0%
Total Ash	3.98	Not exceeding 5.0%
Acid-insoluble Ash	0.06	Not exceeding 1.0%

**Table 2:** Effects of chronic oral administration of the ethanolic seed extract of *M. pruriens* var. *pruriens* on hematological parameters in female rats.

Hematological Parameters	Control	Satellite Control	The Ethanolic Seed Extract of *M. pruriens* var. *pruriens* (mg/kg)			
			100	500	2500	Satellite 2500
RBC (×10^6^/μL)	7.63 ± 0.14	7.96 ± 0.36	7.25 ± 0.30	7.76 ± 0.16	7.65 ± 0.10	7.58 ± 0.04
HB (g/dL)	14.52 ± 0.26	15.00 ± 0.73	13.73 ± 0.55	14.51 ± 0.28	14.48 ± 0.26	14.08 ± 0.05
HCT (%)	46.24 ± 0.88	47.54 ± 2.80	43.27 ± 1.78	46.34 ± 0.95	45.69 ± 0.88	44.52 ± 0.26
MCV (fL)	60.65 ± 1.01	59.66 ± 0.89	59.72 ± 0.35	59.76 ± 0.37	59.66 ± 0.56	58.76 ± 0.07
MCH (pg)	19.05 ± 0.32	18.84 ± 0.17	18.96 ± 0.15	18.70 ± 0.09	18.92 ± 0.18	18.60 ± 0.08
MCHC (g/dL)	31.41 ± 0.15	31.62 ± 0.32	31.74 ± 0.12	31.32 ± 0.12	31.71 ± 0.13	31.62 ± 0.18
PLT (×10^5^/μL)	7.10 ± 0.13	7.10 ± 0.40	6.83 ± 0.22	7.24 ± 0.68	6.85 ± 0.47	7.00 ± 0.30

All values are reported as mean ± S.E.M. (*n* = 10 per group; *n* = 5 per group for satellite groups). RBC, red blood cell; HB, hemoglobin; HCT, hematocrit; MCV, mean corpuscular volume; MCH, mean corpuscular hemoglobin; MCHC, mean corpuscular hemoglobin concentration; PLT, platelet.

**Table 3:** Effects of chronic oral administration of the ethanolic seed extract of *M. pruriens* var. *pruriens* on hematological parameters in male rats.

Hematological Parameters	Control	Satellite Control	The Ethanolic Seed Extract of *M. pruriens* var. *pruriens* (mg/kg)			
			100	500	2500	Satellite 2500
RBC (×10^6^/μL)	8.26 ± 0.11	8.11 ± 0.21	8.05 ± 0.10	8.24 ± 0.06	8.44 ± 0.16	8.51 ± 0.21
HB (g/dL)	15.52 ± 0.18	14.92 ± 0.24	15.09 ± 0.20	15.20 ± 0.12	15.78 ± 0.27	15.64 ± 0.31
HCT (%)	48.91 ± 0.57	47.42 ± 0.96	48.35 ± 0.56	48.80 ± 0.49	50.77 ± 1.24	50.46 ± 1.53
MCV (fL)	59.19 ± 0.44	58.50 ± 0.64	60.09 ± 0.43	59.25 ± 0.43	60.15 ± 0.71	59.20 ± 0.56
MCH (pg)	18.79 ± 0.11	18.42 ± 0.24	18.76 ± 0.11	18.45 ± 0.17	18.71 ± 0.11	18.36 ± 0.10
MCHC (g/dL)	31.74 ± 0.10	31.48 ± 0.15	31.21 ± 0.16	31.14 ± 0.21 *	31.13 ± 0.27 *	31.04 ± 0.36 *
PLT (×10^5^/μL)	7.42 ± 0.11	7.35 ± 0.22	7.62 ± 0.26	7.38 ± 0.21	7.28 ± 0.25	7.49 ± 0.30

All values are reported as mean ± S.E.M. (*n* = 10 per group; *n* = 5 per group for satellite groups). * *p* < 0.05 in comparison to the control group. RBC, red blood cell; HB, hemoglobin; HCT, hematocrit; MCV, mean corpuscular volume; MCH, mean corpuscular hemoglobin; MCHC, mean corpuscular hemoglobin concentration; PLT, platelet.

**Table 4:** Effects of chronic oral administration of the ethanolic seed extract of *M. pruriens* var. *pruriens* on WBC differential counts in female rats.

WBC Differential Counts Parameters	Control	Satellite Control	The Ethanolic Seed Extract of *M. pruriens* var. *pruriens* (mg/kg)			
			100	500	2500	Satellite 2500
WBC (×10^3^/μL)	2.38 ± 0.30	2.53 ± 0.14	2.37 ± 0.06	2.42 ± 0.27	2.38 ± 0.17	3.23 ± 0.48 *
NEU (×10^3^/µL)	0.57 ± 0.11	0.87 ± 0.12	0.59 ± 0.07	0.76 ± 0.09	0.63 ± 0.08	0.91 ± 0.07 *
LYMP (×10^3^/µL)	1.46 ± 0.23	1.45 ± 0.18	1.67 ± 0.30	1.46 ± 0.19	1.37 ± 0.11	1.99 ± 0.32
MONO (×10^3^/µL)	0.26 ± 0.05	0.19 ± 0.06	0.35 ± 0.04	0.17 ± 0.03	0.33 ± 0.07	0.28 ± 0.12
EO (×10^3^/µL)	0.04 ± 0.01	0.04 ± 0.01	0.06 ± 0.01	0.04 ± 0.01	0.05 ± 0.01	0.05 ± 0.01
BASO (×10^3^/µL)	0.00 ± 0.00	0.00 ± 0.00	0.00 ± 0.00	0.00 ± 0.00	0.00 ± 0.00	0.00 ± 0.00

All values are reported as mean ± S.E.M. (*n* = 10 per group; *n* = 5 per group for satellite groups). * *p* < 0.05 in comparison to the control group. WBC, white blood cell; NEU, neutrophil; LYMP, lymphocyte; MONO, monocyte; EO, eosinophil; BASO, basophil.

**Table 5:** Effects of chronic oral administration of the ethanolic seed extract of *M. pruriens* var. *pruriens* on WBC differential counts in male rats.

WBC Differential Counts Parameters	Control	Satellite Control	The Ethanolic Seed Extract of *M. pruriens* var. *pruriens* (mg/kg)			
			100	500	2500	Satellite 2500
WBC (×10^3^/μL)	4.15 ± 0.17	3.70 ± 0.61	3.47 ± 0.36	3.65 ± 0.28	3.94 ± 0.24	3.67 ± 0.28
NEU (×10^3^/µL)	0.79 ± 0.07	0.96 ± 0.17	0.75 ± 0.09	0.98 ± 0.08	0.93 ± 0.08	0.78 ± 0.11
LYMP (×10^3^/µL)	3.01 ± 0.14	2.39 ± 0.45	2.33 ± 0.23 *	2.38 ± 0.22 *	2.76 ± 0.16	2.49 ± 0.18
MONO (×10^3^/µL)	0.28 ± 0.06	0.28 ± 0.08	0.34 ± 0.05	0.24 ± 0.03	0.19 ± 0.04	0.36 ± 0.05
EO (×10^3^/µL)	0.07 ± 0.01	0.06 ± 0.01	0.05 ± 0.01	0.06 ± 0.01	0.06 ± 0.01	0.04 ± 0.02
BASO (×10^3^/µL)	0.00 ± 0.00	0.00 ± 0.00	0.00 ± 0.00	0.00 ± 0.00	0.00 ± 0.00	0.00 ± 0.00

All values are reported as mean ± S.E.M. (*n* = 10 per group; *n* = 5 per group for satellite groups). * *p* < 0.05 in comparison to the control group. WBC, white blood cell; NEU, neutrophil; LYMP, lymphocyte; MONO, monocyte; EO, eosinophil; BASO, basophil.

**Table 6:** Effects of chronic oral administration of the ethanolic seed extract of *M. pruriens* var. *pruriens* on blood chemistry in female rats.

Blood Chemistry Parameters	Control	Satellite Control	The Ethanolic Seed Extract of *M. pruriens* var. *pruriens* (mg/kg)			
			100	500	2500	Satellite 2500
BUN (mg/dL)	20.61 ± 1.16	24.78 ± 1.37 *	22.67 ± 1.30	20.05 ± 0.61	19.18 ± 1.11	25.44 ± 1.07 *
Creatinine (mg/dL)	0.64 ± 0.01	0.51 ± 0.02 *	0.60 ± 0.02	0.65 ± 0.02	0.64 ± 0.01	0.49 ± 0.02 *
Total protein (g/dL)	6.77 ± 0.17	7.44 ± 0.19 *	6.47 ± 0.11	6.42 ± 0.13	6.48 ± 0.08	7.18 ± 0.09
Albumin (g/dL)	3.19 ± 0.06	3.36 ± 0.10	3.19 ± 0.05	3.13 ± 0.05	3.13 ± 0.04	3.20 ± 0.09
Total bilirubin (mg/dL)	0.20 ± 0.02	0.14 ± 0.03 *	0.22 ± 0.01	0.24 ± 0.02	0.19 ± 0.01	0.11 ± 0.01 *
Direct bilirubin (mg/dL)	0.07 ± 0.00	0.06 ± 0.00	0.08 ± 0.00	0.08 ± 0.00	0.13 ± 0.05	0.06 ± 0.00
AST (U/L)	104.90 ± 5.54	115.00 ± 10.02	159.40 ± 26.67 *	126.80 ± 13.77	126.80 ± 14.73	109.40 ± 16.03
ALT (U/L)	47.60 ± 3.54	60.60 ± 3.98	79.10 ± 19.77	52.80 ± 4.68	54.70 ± 11.67	53.20 ± 7.59
ALP (U/L)	67.70 ± 19.22	104.40 ± 22.56	51.60 ± 6.58	50.00 ± 6.42	48.50 ± 6.17	97.80 ± 14.03

All values are reported as mean ± S.E.M. (*n* = 10 per group; *n* = 5 per group for satellite groups). * *p* < 0.05 in comparison to the control group. BUN, blood urea nitrogen; AST, aspartate aminotransferase; ALT, alanine aminotransferase; ALP, alkaline phosphatase.

**Table 7:** Effects of chronic oral administration of the ethanolic seed extract of *M. pruriens* var. *pruriens* on blood chemistry in male rats.

Blood Chemistry Parameters	Control	Satellite Control	The Ethanolic Seed Extract of *M. pruriens* var. *pruriens* (mg/kg)			
			100	500	2500	Satellite 2500
BUN (mg/dL)	16.62 ± 0.59	14.14 ± 0.47 *	14.74 ± 0.40 *	14.20 ± 0.68 *	13.29 ± 0.54 *	13.40 ± 0.58 *
Creatinine (mg/dL)	0.59 ± 0.01	0.58 ± 0.01	0.57 ± 0.02	0.58 ± 0.02	0.55 ± 0.02	0.58 ± 0.02
Total protein (g/dL)	6.55 ± 0.15	6.18 ± 0.07	6.32 ± 0.16	6.44 ± 0.22	6.37 ± 0.22	6.60 ± 0.22
Albumin (g/dL)	2.99 ± 0.07	2.96 ± 0.04	2.89 ± 0.04	2.92 ± 0.06	2.86 ± 0.06	2.94 ± 0.08
Total bilirubin (mg/dL)	0.11 ± 0.01	0.17 ± 0.01	0.15 ± 0.04	0.12 ± 0.01	0.13 ± 0.02	0.18 ± 0.01
Direct bilirubin (mg/dL)	0.06 ± 0.00	0.07 ± 0.01	0.06 ± 0.00	0.06 ± 0.00	0.07 ± 0.00	0.06 ± 0.00
AST (U/L)	117.80 ± 7.01	115.60 ± 12.82	99.90 ± 5.08	112.30 ± 5.63	116.90 ± 11.93	110.00 ± 16.28
ALT (U/L)	50.90 ± 3.74	42.20 ± 4.75	41.90 ± 2.55	44.40 ± 2.30	48.40 ± 4.78	52.20 ± 16.30
ALP (U/L)	72.00 ± 3.78	59.40 ± 6.16	66.70 ± 4.43	83.50 ± 17.59	70.80 ± 8.51	73.60 ± 13.25

All values are reported as mean ± S.E.M. (*n* = 10 per group; *n* = 5 per group for satellite groups). * *p* < 0.05 in comparison to the control group. BUN, blood urea nitrogen; AST, aspartate aminotransferase; ALT, alanine aminotransferase; ALP, alkaline phosphatase.

## Data Availability

The original contributions presented in this study are included in the article/Supplementary Materials. Further inquiries can be directed to the corresponding author.

## Supplementary Materials

The following supporting information can be downloaded at https://www.mdpi.com/article/10.3390/ph19030421/s1, Table S1: Hippocratic screening assessment of female rats in the acute oral toxicity study of the ethanolic seed extract of *M. pruriens* var. *pruriens*; Table S2: Hippocratic screening assessment of female and male rats in the chronic oral toxicity study of the ethanolic seed extract of *M. pruriens* var. *pruriens*.
