# Supplementary material for: Safety Assessment of the Ethanolic Seed Extract of Mucuna pruriens var. pruriens: Acute and Chronic Oral Toxicity Studies in Sprague–Dawley Rats
Source: Pharmaceuticals (Basel). 2026 Mar 4;19(3):421. doi: 10.3390/ph19030421 (PMC13029009; doi:10.3390/ph19030421)
Supplement: Supplementary file 1 [file pharmaceuticals-19-00421-s001.zip › pharmaceuticals-4169773-supplementary.pdf]

Table S1. Hippocratic screening assessment of female rats in the acute oral toxicity study of the ethanolic seed extract of *M. pruriens* var. *pruriens*

**The ethanolic seed extract of *M. pruriens* var. *pruriens* (5000 mg/kg)**	**Hours after drug administration**																							
	1	2	3	4	5	6	7	8	9	10	11	12	13	14	15	16	17	18	19	20	21	22	23	24
Decrease of motor activity	0	0	0	0	0	0	0	0	0	0	0	0	0	0	0	0	0	0	0	0	0	0	0	0
Decrease of respiratory rate	0	0	0	0	0	0	0	0	0	0	0	0	0	0	0	0	0	0	0	0	0	0	0	0
Loss of righting reflex	0	0	0	0	0	0	0	0	0	0	0	0	0	0	0	0	0	0	0	0	0	0	0	0
Loss of screen grip	0	0	0	0	0	0	0	0	0	0	0	0	0	0	0	0	0	0	0	0	0	0	0	0
Time of death (hr.)	-	-	-	-	-	-	-	-	-	-	-	-	-	-	-	-	-	-	-	-	-	-	-	-

Hippocratic screening scores were assigned according to the following scheme. Decrease in motor activity: 0 = no decrease in motor activity, no change in respiratory rate, no loss of righting reflex, no loss of screen grip; +1 = does not move spontaneously, but when handled will move rapidly; +2 = when handled will move slowly; +3 = when handled will move sluggishly; +4 = when handled will not move at all. Decrease in respiration rate: +1 = 10% decrease in respiratory rate; +2 = 20% decrease in respiratory rate; +3 = 40% decrease in respiratory rate; +4 = 80% decrease in respiratory rate. Loss of righting reflex: +1 = can be placed only on one side; +2 = can be placed on either side equally well; +3 = can be placed on the back as well as either side; +4 = cannot be aroused from the back position by the hind leg toe pinch. Loss of screen grip: +1 = rat falls off at first shake of the screen; +2 = rat falls off when the screen has been inverted; +3 = rat falls off when the screen is at a 90° angle; +4 = rat falls off as the screen is tilted to a 45° angle.

Table S2. Hippocratic screening assessment of female and male rats in the chronic oral toxicity study of the ethanolic seed extract of *M. pruriens* var. *pruriens*

**The ethanolic seed extract of *M. pruriens* var. *pruriens* (100, 500, 2500, Satellite 2500 mg/kg)**	**Weeks following drug administration in female rats**																				
	1-2	3-4	5-6	7-8	9-10	11-12	13-14	15-16	17-18	19-20	21-22	23-24	25-26	27-28	29-30	31-32	33-34	35-36	37-38	39-40	41-42
Decrease of motor activity	0	0	0	0	0	0	0	0	0	0	0	0	0	0	0	0	0	0	0	0	0
Decrease of respiratory rate	0	0	0	0	0	0	0	0	0	0	0	0	0	0	0	0	0	0	0	0	0
Loss of righting reflex	0	0	0	0	0	0	0	0	0	0	0	0	0	0	0	0	0	0	0	0	0
Loss of screen grip	0	0	0	0	0	0	0	0	0	0	0	0	0	0	0	0	0	0	0	0	0
Time of death (wk.)	-	-	-	-	-	-	-	-	-	-	-	-	-	-	-	-	-	-	-	-	-
**The ethanolic seed extract of *M. pruriens* var. *pruriens* (100, 500, 2500, Satellite 2500 mg/kg)**	**Weeks following drug administration in male rats**																				
	1-2	3-4	5-6	7-8	9-10	11-12	13-14	15-16	17-18	19-20	21-22	23-24	25-26	27-28	29-30	31-32	33-34	35-36	37-38	39-40	41-42
Decrease of motor activity	0	0	0	0	0	0	0	0	0	0	0	0	0	0	0	0	0	0	0	0	0
Decrease of respiratory rate	0	0	0	0	0	0	0	0	0	0	0	0	0	0	0	0	0	0	0	0	0
Loss of righting reflex	0	0	0	0	0	0	0	0	0	0	0	0	0	0	0	0	0	0	0	0	0
Loss of screen grip	0	0	0	0	0	0	0	0	0	0	0	0	0	0	0	0	0	0	0	0	0
Time of death (wk.)	-	-	-	-	-	-	-	-	-	-	-	-	-	-	-	-	-	-	-	-	-

Hippocratic screening scores were assigned according to the following scheme. Decrease in motor activity: 0 = no decrease in motor activity, no change in respiratory rate, no loss of righting reflex, no loss of screen grip; +1 = does not move spontaneously, but when handled will move rapidly; +2 = when handled will move slowly; +3 = when handled will move sluggishly; +4 = when handled will not move at all. Decrease in respiration rate: +1 = 10% decrease in respiratory rate; +2 = 20% decrease in respiratory rate; +3 = 40% decrease in respiratory rate; +4 = 80% decrease in respiratory rate. Loss of righting reflex: +1 = can be placed only on one side; +2 = can be placed on either side equally well; +3 = can be placed on the back as well as either side; +4 = cannot be aroused from the back position by the hind leg toe pinch. Loss of screen grip: +1 = rat falls off at first shake of the screen; +2 = rat falls off when the screen has been inverted; +3 = rat falls off when the screen is at a 90° angle; +4 = rat falls off as the screen is tilted to a 45° angle.
